# The trajectory of gray matter development in Broca’s area is abnormal in people who stutter

**DOI:** 10.3389/fnhum.2015.00089

**Published:** 2015-03-03

**Authors:** Deryk S. Beal, Jason P. Lerch, Brodie Cameron, Rhaeling Henderson, Vincent L. Gracco, Luc F. De Nil

**Affiliations:** ^1^Department of Communication Sciences and Disorders and the Institute for Stuttering Treatment and Research, Faculty of Rehabilitation Medicine, University of AlbertaEdmonton, AB, Canada; ^2^Neuroscience and Mental Health Institute, University of AlbertaEdmonton, AB, Canada; ^3^Program in Neuroscience and Mental Health, The Hospital for Sick ChildrenToronto, ON, Canada; ^4^Department of Medical Biophysics, University of TorontoToronto, ON, Canada; ^5^Haskins LaboratoriesNew Haven, CT, USA; ^6^Centre for Research on Brain, Language and Music, McGill UniversityMontreal, QC, Canada; ^7^Department of Speech-Language Pathology, University of TorontoToronto, ON, Canada

**Keywords:** developmental stuttering, Broca’s area, speech production, motor control, cortical thickness, neurodevelopment, inferior frontal gyrus, developmental disorders

## Abstract

The acquisition and mastery of speech-motor control requires years of practice spanning the course of development. People who stutter often perform poorly on speech-motor tasks thereby calling into question their ability to establish the stable neural motor programs required for masterful speech-motor control. There is evidence to support the assertion that these neural motor programs are represented in the posterior part of Broca’s area, specifically the left pars opercularis. Consequently, various theories of stuttering causation posit that the disorder is related to a breakdown in the formation of the neural motor programs for speech early in development and that this breakdown is maintained throughout life. To date, no study has examined the potential neurodevelopmental signatures of the disorder across pediatric and adult populations. The current study aimed to fill this gap in our knowledge. We hypothesized that the developmental trajectory of cortical thickness in people who stutter would differ across the lifespan in the left pars opercularis relative to a group of control participants. We collected structural magnetic resonance images from 116 males (55 people who stutter) ranging in age from 6 to 48 years old. Differences in cortical thickness across ages and between patients and controls were investigated in 30 brain regions previously implicated in speech-motor control. An interaction between age and group was found for the left pars opercularis only. In people who stutter, the pars opercularis did not demonstrate the typical maturational pattern of gradual gray matter thinning with age across the lifespan that we observed in control participants. In contrast, the developmental trajectory of gray matter thickness in other regions of interest within the neural network for speech-motor control was similar for both groups. Our findings indicate that the developmental trajectory of gray matter in left pars opercularis is abnormal in people who stutter.

## Introduction

Stuttering is a complex developmental communication disorder affecting 1% of the population (Yairi and Ambrose, 1999; Boyle et al., 2011). Although there have been recent advances in our understanding of the genetic and neural influences on stuttering, the etiology and pathophysiology of the disorder remain poorly defined. The speech characteristics associated with the disorder are proposed to be the result of an aberrant brain mechanism caused by the interaction of genetic and environmental variables (De Nil, 1999; Ludlow and Loucks, 2003; Bohland et al., 2014). These characteristics, namely speech sound repetitions, prolongations and silent blocks, typically first present in affected children between the ages of 18 and 60 months (Yairi and Ambrose, 1999). Although 74% of children recover from stuttering within 4 years of onset, 26% persist to stutter over their lifespan and experience a resultant negative impact on their quality of life (Yairi and Ambrose, 1999; Craig et al., 2009).

Magnetic resonance imaging studies of brain structure in children and adults who stutter have begun to elucidate the role of neuroanatomical abnormalities in the disorder. Importantly, children and adults who stutter do not demonstrate gross abnormalities in total brain volume or overall gray matter, white matter or cerebral spinal fluid volumes (Beal et al., 2007, 2013; Chang and Zhu, 2013). Rather, the observed abnormalities in gray and white matter are regional in nature and, for the most part, localized to the parasylvian and subcortical regions known to be important for speech perception and production (Sommer et al., 2002; Jäncke et al., 2004; Beal et al., 2007, 2013; Chang et al., 2008; Watkins et al., 2008; Chang and Zhu, 2013).

A common finding that is emerging from the developmental stuttering literature is the repeated implication in the disorder of microstructural abnormalities in the gray and white matter regions that comprise Broca’s area. Broca’s area is defined, via cytoarchitectonic mapping, as Brodmann’s areas (BA) 44 and 45. BA44 and 45 are relatively synonymous with the pars opercularis and pars triangularis of the left hemisphere which are labeled via the cortical folding nomenclature (Amunts et al., 1999; Lindenberg et al., 2007; Keller et al., 2009; Amunts and Zilles, 2012; Lemaire et al., 2013). Modern models of the neural network for speech production posit that neurons in the left pars opercularis code for speech sounds and that the region is critically important for both speech acquisition and mature speech production (Guenther et al., 1998; Hickok et al., 2011; Guenther and Vladusich, 2012; Hickok, 2012). Neural representations of speech sounds and their sensorimotor processes are likely acquired via the support of a bilateral, but left hemisphere biased, neural network that is shared for speech perception and production (Guenther, 1995; Bailly, 1997; Guenther and Vladusich, 2012). The sensory component of the sensorimotor network is proposed to map acoustic speech signals to articulatory representations (Hickok and Poeppel, 2004, 2007; Guenther, 2006; Tourville et al., 2008). The major anatomical structures that comprise this afferent pathway are the primary auditory cortex, temporoparietal junction and pars opercularis of the inferior frontal gyrus. The motor component of the sensorimotor network is comprised of the pars opercularis, premotor and primary motor cortices and the subsequent cortico-spinal tracts. As such it is unsurprising that various studies have identified neuroanatomic abnormalities in these regions in people who stutter with the relatively consistent inclusion of the left pars opercularis of Broca’s area.

Of recent interest are the findings of neuroanatomic abnormalties in young children who stutter as they provide a window into brain structure much closer in time to the onset of the disorder than the studies focused on adults alone. Children who stutter have been shown to have less gray matter volume in the left and right inferior frontal gyri relative to controls (Chang et al., 2008; Beal et al., 2013). Specifically, in the left inferior frontal gyrus children who stutter are known to have less gray matter volume in the pars opercularis, pars triangularis and pars orbitalis. In the right inferior frontal gyrus children who stutter have less gray matter volume in the pars opercularis and pars triangularis. Beal et al. (2013) found that gray matter volume in the right pars triangularis and opercularis was negatively correlated with stuttering severity supporting the premise that more typical development in these regions aids compensation for stuttered speech. Children who stutter as young as 3 years of age have been shown to have less structural connectivity of white matter originating in the region of the left pars opercularis that link it to the left posterior temporal areas via the insula, putamen and extreme capsule as well as less connectivity between the right pars opercularis and the putamen (Chang and Zhu, 2013). The finding of less structural connectivity between the left pars opercularis and other regions of the neural network for speech production, including sensory regions, is consistent with previous studies of white matter properties of both children and adults who stutter that reported lesser fractional anisotropy (FA) values in white matter generally along the superior longitudinal fasciculus underlying the broader gray matter regions proximate to, and including, the left pars opercularis (Sommer et al., 2002; Chang et al., 2008; Watkins et al., 2008; Cykowski et al., 2010). Furthermore, the white matter underlying regions adjacent to the left pars opercularis, namely the left ventral premotor and middle primary motor cortices, has been noted to be less well connected to other speech relevant brain regions in adults who stutter vs. controls (Cai et al., 2014b; Connally et al., 2014).

There is a relatively larger literature that has examined adults who stutter and found that they too present with neuroanatomic abnormalities in parasylvian regions as compared to controls with regards to regional gray matter volume as measured by voxel-based morphometry. However, the directionality of the results reported in the literature is inconsistent. Specifically, adults who stutter have been found to have more gray matter volume in the left inferior frontal gyrus as well as the bilateral pre and post central gyri, superior temporal gyri, middle temporal gyri, basal ganglia and cerebellum (Beal et al., 2007; Lu et al., 2010). Conversely, adults who stutter have also been found to have less gray matter volume in the left inferior frontal gyrus, left superior frontal gyrus as well as the bilateral middle frontal gyri, cerebellar posterior lobes, dorsal part of medulla and the cerebellar tonsil (Kell et al., 2009; Lu et al., 2010). Interestingly, not all studies have found differences in gray matter volume in adults who stutter (Jäncke et al., 2004). Further study of the neuroanatomy of adults who stutter is needed to clarify just how different the cortical structures pertinent to speech acquisition and production are relative to controls.

A number of parasylvian brain regions have been implicated in stuttering but it has been difficult to determine which regions are critical to the development of the disorder. Based on the common finding of gray and white matter structural abnormalities in and around the left pars opercularis, many authors have proposed that developmental stuttering is the result of a breakdown in motor control of articulation at the cortical level around this region (Sommer et al., 2002; Max et al., 2004; Brown et al., 2005; Watkins et al., 2008; Kell et al., 2009). Specifically, some researchers have proposed that stuttering may result from a failure to correctly form the neural representations of speech sounds during development due to difficulties with the integration of auditory or somatosensory information with said representations (Neilson and Neilson, 1987; Max et al., 2004; Corbera et al., 2005; Weber-Fox et al., 2008; Beal et al., 2013). In typically developing children between 5–12 years of age, the gray matter comprising the left inferior frontal gyrus and bilateral posterior superior temporal gyri continues to thicken over this short time span when the majority of other brain regions have started a processes of thinning (Sowell et al., 2004). This unique pattern of development suggests that there may be a special relationship between gray matter maturation and the mastery of speech-motor skills over this age span. Although the maturation of speech and language regions appears to be protracted relative to other brain regions, the overall pattern across adulthood is one of gradual gray matter thinning (Sowell et al., 2003) Despite the fact that stuttering is often a lifelong affliction, we are unaware of any study that has examined the potential neurodevelopmental signatures of the disorder across a pediatric and adult population of people who stutter. The current study aimed to fill this gap in the knowledge base and provide further insight into the regions that may be critically important for developmental stuttering by examining the trajectory of neurodevelopment in various speech relevant regions including Broca’s area as defined by the pars oeprcularis and pars triangularis.

Our investigation of the interaction of aging and group effects for cortical thickness aimed to identify regions that are problematic in their development over the lifespan in people who stutter. To describe the developmental differences in gray matter thickness in these regions we conducted a mega-analysis of a large cohort of children and adults who stutter utilizing magnetic resonance images of the brain collected across various smaller studies. If the onset and maintenance of stuttering is related to deficient neural resources for establishing the internal representations of speech-motor programs associated with the pars opercularis than neurodevelopment of this region should be implicated in the disorder. Based on our above review of the literature, we hypothesized that the developmental trajectory of cortical thickness in the left pars opercularis would be abnormal across the lifespan in people who stutter. To investigate whether or not development of this region played a key role in the disorder we tested the developmental trajectory of a total of 30 parasylvian regions known to contribute to the sensory-motor interactions that are associated with speech acquisition.

## Materials and methods

### Participants

Participants were 116 right-handed males with English as their primary language and a normal developmental and medical history. The 55 boys and men with persistent developmental stuttering ranged in age from 7 to 47 years (mean = 26.07, median = 27, s.d. = 10.79) and the 61 matched control participants from 6 to 48 years (mean = 26.79, median = 27, s.d. = 10.10). The two groups did not differ in age (*t*_(110)_ = 0.37 *p* = 0.71). The Stuttering Severity Index—3rd Edition was used to measure stuttering severity in the patient group. Stuttering severity ranged from very mild (5) to very severe (49) (mean = 21.80, median = 21, s.d. = 8.92). One patient’s stuttering severity score was not available due to technical difficulty.

### MRI acquisition

T1 images for 51 participants (23 people who stutter) were collected on a 1.5-T MRI system (GE Medical Systems, Milwaukee, Wisconsin, USA) using a standard quadrature head coil at the Toronto Western Hospital in Toronto, Ontario, Canada. A T1-weighted three-dimensional inversion recovery-prepared fast spoiled gradient echo (FSPGR) sequence (flip angle = 20 degrees, echo time (TE) = 5.2 ms, repetition time (TR) = 12 ms, preparation time = 300 ms) was used to generate 124 1.5 mm-thick sagittal slices (256 × 256 matrix). T1 images for 47 participants (23 people who stutter) were collected on a 1.5-T MRI system at the Hospital for Sick Children in Toronto, Ontario, Canada. A 1.5-T Signa Excite HD 12.0 MRI System (GE Medical Systems, Milwaukee, Wisconsin, USA) with an 8-channel head coil was used to obtain neuroantomic images. A T1-weighted 3D FSPGR sequence (flip angle = 9 degrees, TE = 4.2 ms, TR = 9 ms) was used to generate 114 1.5 mm-thick axial slices (256 × 192 matrix, 24 cm field of view). Pixels were squared post processing ultimately resulting in a 256 × 256 viewing matrix. The images for the remaining 18 participants (9 people who stutter) were collected on a 3-T MRI system (Siemens Medical Solutions) using an 8-channel head coil at the Magnetic Resonance Research Center at Yale University in New Haven, Connecticut, USA. A T1-weighted three-dimensional MPRAGE sequence (TE = 3.7 ms, TR = 2.5 ms) was used to generate 176 1 mm-thick sagittal slices (256 × 256 matrix).

### MRI preprocessing

**Figure 1:**
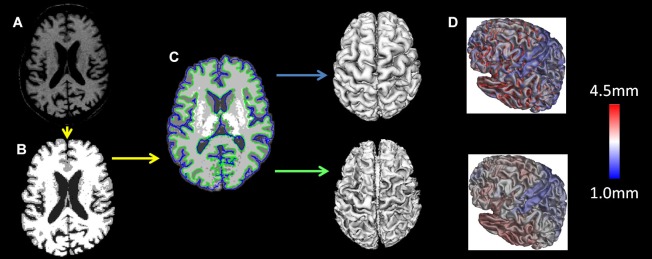
**MRI preprocessing:** the T1 images were **(A)** registered to the ICBM 152 template with a 12-parameter linear transformation, **(B)** RF inhomogeneity corrected and skull stripped, **(C)** tissue segmented and gray and white matter surfaces created and **(D)** tissue partial volumes estimated. Cortical thickness was measured as the distance between the white and gray surfaces in native space.

T1 images were registered to the ICBM 152 template with a 12-parameter linear transformation (Collins et al., 1994), RF inhomogeneity corrected (Sled and Pike, 1998), skull stripped (Smith, 2002), tissue classified (Zijdenbos et al., 2002) and tissue partial volume estimated (Tohka et al., 2004). Deformable models were used to fit the white matter surface for each hemisphere separately, followed by an expansion outward to find the gray matter/CSF intersection (MacDonald et al., 2000; Kim et al., 2005) resulting in 4 surfaces of 41,962 polygons each. From these surfaces the distance between the white and gray surfaces in native space was used to measure cortical thickness (Lerch and Evans, 2005). The individual surfaces were non-linearly aligned to the ICBM 152 template using surface-based registration techniques (Robbins et al., 2004). All analyses were performed using native cortical thickness. The preprocessing steps are depicted in Figure 1.

### Region of interest analysis

**Figure 2:**
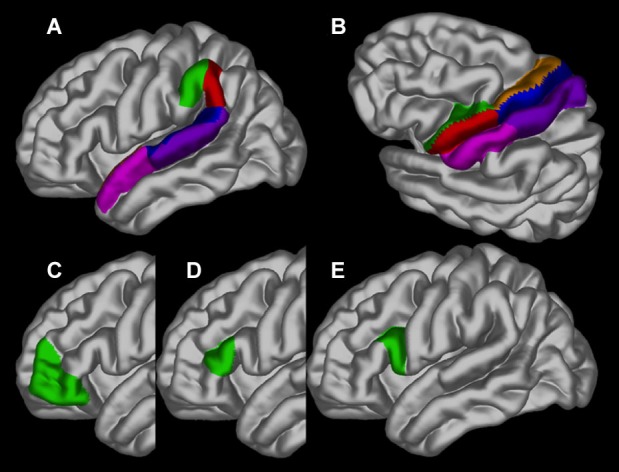
**The regions of interest depicted on the gray matter surface of the left hemisphere: (A)** the anterior (green) and posterior (red) angular gyrus, anterior (pink) and posterior (blue) superior temporal sulcus and anterior (dark pink) and posterior (blue) superior temporal gyrus; **(B)** ventral (green) and dorsal (brown) premotor cortex, ventral (red) and dorsal (blue) primary motor cortex and ventral (pink) and dorsal (purple) sensorimotor cortex; **(C)** pars orbitalis (BA47); **(D)** pars triangularis (BA45) and **(E)**. pars opercularis (BA44).

Differences in cortical thickness across ages between people who stutter and controls were investigated in 30 specific brain regions of interest (ROI), separately for each hemisphere, based on our hypothesis. The ROIs are depicted in Figure 2 and consisted of the following in each hemisphere: pars opercularis (BA44), pars triangularis (BA45), pars orbitalis (BA47), ventral and dorsal premotor cortex, ventral and dorsal primary motor cortex, ventral and dorsal sensorimotor cortex, anterior and posterior angular gyrus, anterior and posterior superior temporal sulcus and anterior and posterior superior temporal gyrus. Using the average gray surface of all participants the ROI delineations were back projected onto the individual surfaces using the surface transformation and the average ROI thickness computed for each individual. For each ROI a linear mixed effect model was calculated with the intercept allowed to vary between scanners to correct for possible scanner effects and a False Discovery Rate was used (Genovese et al., 2002).

## Results

**Figure 3:**
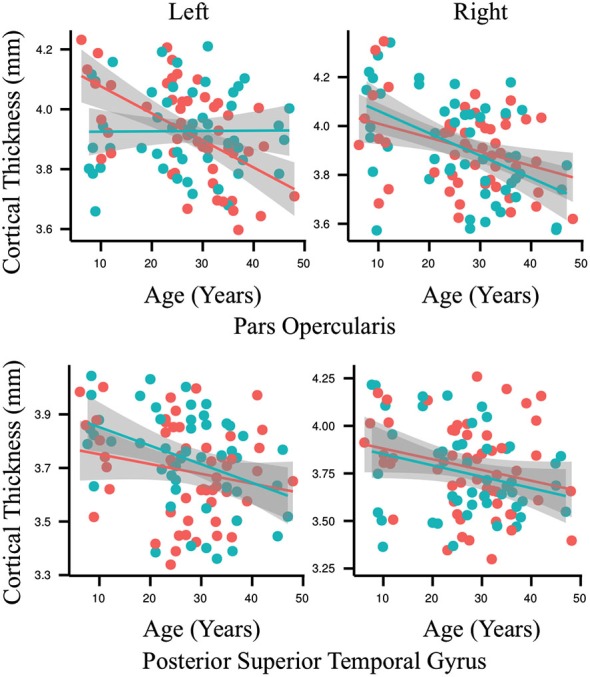
**Each graph depicts the association between age (6–49 years old) and cortical thickness (millimeters) for an exemplary region interest**. Results for the people who stutter are shown in red and for the control group in blue. Regression lines are included and circles indicate individual measurements. The only significant interaction between age and group for cortical thickness was found in the left pars opercularis (*p* = 0.001, *q* = 0.04). By contrast, the developmental trajectory of thickness for all of the other ROIs was similar for both groups and no statistically significant differences were identified.

The results of the ROI statistical analyses are shown in Table 1. As expected, there was a main effect for age in nearly every ROI. Only the left anterior superior temporal gyrus and right anterior superior temporal sulcus did not demonstrate an effect for age. As can be seen in Figure 3, gray matter cortex was thickest in childhood and then thinned with age in nearly every ROI. There was no main effect for group in any of the ROIs. A significant interaction between age and group was found for the left pars opercularis (*F* = 10.63, *p* = 0.001, *q* = 0.04). Gray matter in this region was thinner in the younger people who stutter relative to their same aged peers but this association inversed with age such that gray matter was thicker in older people who stutter relative to their same age peers. By contrast, the developmental trajectory of thickness for the other ROIs was similar for both groups and no statistically significant interactions were identified.

**Table 1:** **Age, group and interaction results for each region of interest**.

	Age		Group		Interaction		
Region	*F*	*p*	*F*	*p*	*F*	*p*	*q*
**Left hemisphere**
Pars orbitalis	6.05	0.02	0.003	0.95	0.13	0.72	0.94
Pars triangularis	24.11	<0.00001	0.14	0.71	1.41	0.24	0.89
Pars opercularis	7.10	<0.01	0.65	0.42	10.63	0.001	0.04
Ventral premotor cortex	5.34	0.02	0.32	0.57	1.36	0.25	0.89
Dorsal premotor cortex	14.20	<0.001	0.30	0.58	0.45	0.51	0.89
Ventral primary motor cortex	15.16	<0.001	0.38	0.54	0.45	0.51	0.89
Dorsal primary motor cortex	18.53	<0.0001	0.001	0.97	1.96	0.16	0.89
Ventral sensorimotor cortex	21.77	<0.00001	0.002	0.96	0.76	0.39	0.89
Dorsal sensorimotor cortex	30.30	<0.0000001	0.09	0.77	0.32	0.57	0.89
Anterior angular gyrus	11.75	<0.001	0.002	0.97	0.44	0.51	0.89
Posterior angular gyrus	26.94	<0.000001	0.06	0.80	0.03	0.86	0.94
Anterior superior temporal sulcus	5.78	0.02	0.20	0.66	2.87	0.09	0.89
Posterior superior temporal sulcus	21.88	<0.00001	0.09	0.76	0.01	0.92	0.94
Anterior superior temporal gyrus	0.06	0.81	0.52	0.47	1.24	0.27	0.89
Posterior superior temporal gyrus	11.20	0.001	0.99	0.32	0.75	0.39	0.89
**Right hemisphere**
Pars orbitalis	6.82	0.01	0.23	0.63	0.01	0.94	0.94
Pars triangularis	16.22	0.0001	1.80	0.18	1.10	0.30	0.89
Pars opercularis	16.78	<0.0001	0.12	0.74	0.01	0.92	0.94
Ventral premotor cortex	20.85	0.00001	0.06	0.80	0.09	0.77	0.94
Dorsal premotor cortex	16.10	0.0001	0.001	0.98	0.07	0.79	0.94
Ventral primary motor cortex	10.08	<0.001	1.92	0.17	0.52	0.47	0.89
Dorsal primary motor cortex	25.43	<0.00001	0.30	0.59	1.63	0.20	0.89
Ventral sensorimotor cortex	27.05	<0.000001	0.25	0.62	0.02	0.88	0.94
Dorsal sensorimotor cortex	26.46	<0.00001	0.15	0.70	0.37	0.54	0.89
Anterior angular gyrus	9.93	<0.01	0.65	0.42	0.05	0.82	0.94
Posterior angular gyrus	8.37	<0.01	0.00003	1.00	0.29	0.59	0.89
Anterior superior temporal sulcus	0.87	0.35	0.19	0.66	0.15	0.70	0.94
Posterior superior temporal sulcus	7.95	<0.01	0.30	0.59	0.32	0.57	0.89
Anterior superior temporal gyrus	2.23	0.01	0.73	0.39	0.49	0.48	0.89
Posterior superior temporal gyrus	9.22	<0.01	0.0005	0.98	0.33	0.57	0.89

*False discovery rate was used to correct for multiple comparisons (q)*.

## Discussion

Our cross-sectional study is the first to examine the maturational time course of cortical gray matter thickness associated with persistent developmental stuttering across the lifespan in a large cohort of patients. Our data indicate that the developmental trajectory of gray matter thickness in Broca’s area, specifically the left pars opercularis, is abnormal in people who stutter. Within the group of people who stutter, the gray matter in the left pars opercularis did not demonstrate the typical linear maturational pattern of gradual life-long thinning associated with the controls but rather was unchanging across the lifespan. No other regional group differences were identified. Our findings suggest that the neuroanatomic microstructure of the left pars opercularis is crucially important for the development of fluent speech-motor control and that deviations in the development of this region are associated with the onset and persistence of stuttered speech.

The acquisition of speech-motor control spans the course of infant, child and adolescent development (Green et al., 2002; Walsh and Smith, 2002; Smith and Zelaznik, 2004; Nip et al., 2009). Modern models of speech production assert that the pars opercularis is a critical node in the neural network for speech learning (Guenther et al., 1998; Bohland and Guenther, 2006; Hickok and Poeppel, 2007; Hickok et al., 2011; Guenther and Vladusich, 2012; Hickok, 2012). The neurons within the left pars opercularis are active when listening to and producing speech sounds suggesting that they both are crucial to acquiring and maintaining the neural representations of speech-motor programs associated with this region (Devlin and Watkins, 2007; Möttönen et al., 2013). This assertion is supported by a recent meta-analysis of functional neuroimaging experiments that found that imitation and speech have a common neural basis within Broca’s area with an emphasis on the pars opercularis (Kühn et al., 2013). The flat and unchanging linear pattern of gray matter maturation in the left pars opercularis in people who stutter may be reflective of a failure of neural proliferation and dendritic expansion that is critical to support the acquisition and mastery of speech-motor control at a crucial period of childhood speech development. This appears to be followed by a failure, in adolescence and adulthood, of the synaptic pruning process that results in an inefficient neural organization of the resources in this region that are necessary for masterful and fluent speech production. It is particularly interesting that natural recovery from stuttering is very rare once adolescence is reached and that the closing of a window for natural recovery overlaps with this key period where pruning of cortical gray matter, in typically developing adolescents and adults, leads to improved efficiency of networks of neural regions for various tasks.

Our observed abnormal developmental pattern may contribute to the chronic nature of stuttering, as adult-like efficiency of neural processing in the pars opercularis is not attained with maturation. There is an emerging literature of behavioral evidence to support this assertion. Children who stutter as young as 4-years-old are known to have abnormal speech-planning and execution skills as evidenced by higher variability in oral-motor coordination relative to typically developing peers (Smith et al., 2012). People who stutter continue to show higher variability in speech-motor movements than controls in adulthood (Kleinow and Smith, 2000; Smith and Kleinow, 2000). Adults who stutter also have difficulty learning speech-motor, as well as non-speech motor, sequences compared to controls (Smits-Bandstra et al., 2006) and adapt poorly to perturbations in auditory and tactile sensory feedback important for online speech-motor control (Neilson and Neilson, 1987; Loucks and De Nil, 2006a,b; Loucks et al., 2007; Namasivayam and van Lieshout, 2008; Cai et al., 2012, 2014a).

Importantly, there is a precedent for associating changes in cortical gray matter development with speech-motor and language related behaviors. The pars opercularis and posterior superior temporal gyrus show protracted development relative to other brain regions over the school-age years suggesting the importance of continued cortical maturation in these areas for speech-motor and language development during this period (Sowell et al., 2004). The thinning of frontal gray matter during childhood is correlated with improved verbal learning between the ages of 7–16 years (Sowell et al., 2001). Furthermore, reductions of gray matter density in the left dorsolateral frontal and lateral parietal regions during adolescence are correlated with improved vocabulary scores and better performance during verbal learning tasks (Lee et al., 2007). Improved motor skills are also associated with thinning in more dorsal regions of frontal cortex (Lu et al., 2007). Preschool children with apraxia of speech demonstrated thicker left supramarginal gyri than controls and a thinning of the left posterior superior temporal gyrus over a short course of speech therapy (Kadis et al., 2014). As such, gray matter maturation is important for normal speech, language and motor skill development.

Our control data demonstrate the typical pattern of gray matter development defined by thicker gray matter cortex in pre-adolescent followed by a steady and consistent post-adolescent decrease across the majority of neural regions for the remainder of life (Figure 3, red; Sowell et al., 2003, 2004). Early in childhood, progressive gray matter growth observed via MRI is attributed to neural proliferation and dendritic expansion. As the brain matures new connections are made between neurons to facilitate development, for example, the acquisition of motor skills (Draganski et al., 2004, 2006). In contrast to the rapid cortical thickening that occurs in childhood, starting in early adolescence maturation leads to thinning of gray matter by way of synaptic pruning (Sowell et al., 2001). Unlike dendritic expansion, synaptic pruning is a regressive process that creates a more efficient neural structure through the reduction or elimination of potentially unnecessary neural connections and cells. This removal of weaker or neglected cells and connections is thought to improve the efficiency of the remaining neuronal signals. Thinning of the cortex continues to some degree for the remainder of the lifespan and this pattern is observable in our control data (Figure 3, red). The people who stutter also demonstrate this pattern in all of the speech ROI examined except for the left pars opercularis (Figure 3, blue).

The identification of a developmental pathology within the left pars opercularis in people who stutter is consistent with the existing structural and functional neuroimaging literature and modern hypotheses of stuttering onset and persistence. At this time it is impossible to determine if the deficient development of gray matter within the left pars opercularis is causal or the result of the repeatedly documented limited white matter connectivity that this region has with critical nodes within the neural network for speech production in both children and adults who persistent phenotypes of the disorder (Sommer et al., 2002; Chang et al., 2008, 2011; Cykowski et al., 2010; Chang and Zhu, 2013). It is reasonable to suggest that these structural abnormalities in Broca’s area are related to the deficient neural activity observed during speech in both children and adults who stutter. Lesser brain activity has been noted in the left pars opercularis and overlapping the white matter pathways found to be deficient in adults who stutter (Watkins et al., 2008). Furthermore, children and adults who stutter have been noted to have neural timing and sequencing errors directly related to functioning of the left pars opercularis and regions known to be closely connected to it, such as the superior temporal gyrus, relative to controls during speech processing tasks as measured by magnetoencephalography (Salmelin et al., 2000; Beal et al., 2010, 2011). Various functional magnetic resonance and positron emission studies have shown that adults who stutter have increased neural activity in the right hemisphere homologues of parasylvian regions during speech relative to controls (Fox et al., 1996; Brown et al., 2005; De Nil et al., 2008) and that this activity is normalized with treatment and recovery (De Nil et al., 2003; Neumann et al., 2005; Kell et al., 2009). Many authors have attributed these observations to neural compensation for a left inferior frontal deficit (De Nil, 1999; Ludlow and Loucks, 2003; Brown et al., 2005; Kell et al., 2009) that results in faulty neural motor commands for speech production (Max et al., 2004; Civier et al., 2010). Our data demonstrate, for the first time, a lifelong microstructural deficit in the left pars opercularis in a large sample of people with persistent developmental stuttering thereby lending credence to the notion that it plays a role in the cause of the disorder and that right hemisphere biased brain activity noted in adults who stutter is indeed compensatory. Future studies examining both gray and white matter development in childhood stuttering should employ a large cohort followed longitudinally over the seemingly critical age range for speech-motor development.

## Conflict of interest statement

The authors declare that the research was conducted in the absence of any commercial or financial relationships that could be construed as a potential conflict of interest.
